# Emotional Content of Social Representations and Interpersonal Communication

**DOI:** 10.5334/irsp.919

**Published:** 2025-08-14

**Authors:** Pascal Moliner, Anthony Piermattéo

**Affiliations:** 1 Epsylon-LAPECS EA4556, Universitéde Montpellier Paul Valéry, F-34000 Montpellier, FR; 2 PSyCOS –ETHICS EA7446, UniversitéCatholique de Lille, F-59000 Lille, FR

**Keywords:** emotion, social representation, social sharing of emotion

## Abstract

Social representations (SRs) emerge from interactions among members of a group. These interactions enable individuals to share beliefs, build consensus, and maintain shared understandings. While interpersonal communication may initially be driven by the unfamiliarity of SR objects, it can also be motivated by the need for group members to cognitively process these objects when they are perceived as threatening or problematic. Thus, emotions elicited by an object in the social environment may prompt interpersonal communication.

Two studies were conducted to test this hypothesis using online questionnaires. The first study (*N* = 294) revealed a correlation between the emotional tone of individuals’ content regarding the SR of AIDS and the number of people with whom they discussed the topic. The second study, focusing on the SR of war (*N* = 246), confirmed these results. It also showed that social sharing related to “war” as an SR object is mediated by epistemic motivation.

These findings are interpreted from a dual perspective: SR theory and the social sharing of emotions paradigm. They suggest that when a SR object evokes emotions, those emotions, in turn, drive the motivation to engage in social sharing.

Emotions can be defined as “episodic, relatively short-term, biologically based patterns of perception, experience, physiology, action, and communication that occur in response to specific physical and social challenges and opportunities” (Keltner & Gross, 1999, p. 468). When individuals experience an emotional episode, they enter a state of “remanence”, which can last from several hours to several weeks, depending on the intensity of the emotion (Rimé, 1989). This state has cognitive (e.g., rumination, intrusive thoughts), emotional (e.g., reactivation of the emotion each time the episode is recalled), and social (e.g., the need to talk about the emotional episode) consequences.

The social sharing of emotions (Rimé, 2005) arises from these social consequences of the state of persistence. It can be defined as the re-evocation of an emotional episode in a socially shared language, which implies “the presence, at least at a symbolic level, of a partner to whom this re-evocation is addressed” (Rimé, 2005, p. 86). This process occurs in 80% to 95% of cases and facilitates the interpersonal transmission of emotion (Christophe & Rimé, 1997).

Moreover, as Rimé (2020) emphasizes, social sharing can also contribute to the construction of meaning about the world around us. He cites SR theory (Moscovici, 1961) as one theoretical framework capable of accounting for this meaning-making process. In other words, Rimé suggests that the social sharing of emotions may play a role in the formation of SRs. However, he concludes by stressing the need for empirical data to support this hypothesis.

From our perspective, one of the first questions such data should address is the following: Does the emotion elicited by an SR object lead to a process of social sharing? According to Moscovici’s theory, the formation of a SR results from social interactions within a group. Drawing on Rimé’s work on emotional sharing, we may hypothesize that such interactions will occur more frequently when the object of representation evokes strong emotions in individuals. In other words, within the framework of SR theory, if the social sharing of emotions “contributes to the construction of meaning,” it does so primarily by promoting social interactions. The aim of the present research is therefore to provide empirical evidence supporting the hypothesis that the more emotion a SR object elicits, the more frequent the social interactions surrounding that object will be.

In this regard, previous research has shown that the mental evocation of an SR object is likely to trigger emotional responses (e.g., Deschamps & Guimelli, 2002; Tavani & Collange, 2017). Consequently, we can expect that the state of remanence induced by such evocation will lead to social sharing. In other words, the more an SR object arouses emotion, the more individuals are likely to feel the need to share that emotional experience with others.

Indeed, studies have demonstrated a significant correlation between the intensity of emotion experienced and the frequency of social sharing (Delfosse et al., 2004; Luminet et al., 2000). As a result, we should expect to observe more frequent interpersonal communication among individuals for whom the SR object evokes strong emotions, compared to those for whom it provokes weaker or no emotional response.

## Social Representations Theory

Inspired by Durkheim (1898), the SRs theory was introduced by Moscovici (1961, 2008). Moscovici initially defined SRs as “universes of opinions” about objects in the social environment. Later, Abric (1987) expanded on this definition, describing SRs as “the product and process of a mental activity by which an individual or a group reconstructs the reality with which it is confronted and gives it a specific meaning” (p. 64). According to Abric (1994, p. 13), SRs are “a system of interpretation of reality that governs the relationship of individuals to their physical and social environment and determines their behavior or practices.” He further emphasizes that SRs serve as guides for action, directing behaviors and social relationships. They function as a system for pre-decoding reality, shaping expectations and anticipations. SRs allow individuals and groups to interpret and make sense of their social world. They can be understood as “grids for reading the environment” (Moliner, 1992), providing frameworks for perception, analysis, and judgment.

Empirically, SRs appear as sets of information, opinions, or beliefs shared by members of a group regarding objects in their social environment (Moliner et al., 2002). These objects can vary widely in nature—they may be situations, practices, natural phenomena, or even individuals. Regardless of type, these objects are central to social interaction because they either help structure the social order (e.g., work, money, family) or pose a threat to it (e.g., illness, unemployment, climate risks). In other words, they are socially invested objects, which often gives them an ambiguous, multifaceted, or even contentious character (Moliner, 1993). In general, studies on SRs show that they accommodate competing definitions within society (Mouret et al., 2013; Tavani et al., 2021). Different social groups may therefore develop distinct representations of the same object.

Since SR theory has already been extensively reviewed elsewhere (e.g., Rateau et al., 2011), we will not provide a detailed overview here. Instead, we focus on a key aspect of the theory: for most scholars, SRs are viewed as the outcome of interactions among members of a group concerning a particular object (e.g., Lefevre & Lefevre, 2014; Marková, 2003; Morgan, 2009; Rose & Winther-Lindqvist, 2021). These interactions enable individuals to share beliefs, build consensus, and sustain shared meanings. As such, interpersonal communication within a group plays a central role in the formation and maintenance of SRs. Initially, such communication may be driven by the unfamiliarity of the object in question (Moscovici, 1961, 2008). It may also arise from a need for group members to cognitively appropriate an object perceived as threatening or problematic. However, as some studies suggest (Bouriche, 2014, 2022; Guimelli & Rimé, 2009; Rimé, 2005; Piermattéo, 2013), these interactions may also be motivated by the emotions that an object in the social environment elicits within the group.

## Verbal Production and Emotions

A growing body of research demonstrates that mentally evoking an object, situation, or event is likely to elicit emotions associated with that mental imagery (Holmes & Mathews, 2005; Holmes et al., 2008; Ji et al., 2016; Mathews et al., 2013; Suess & Rahman, 2015). Generally, the emotional valence—positive or negative—of the evoked content determines the valence of the emotions experienced. This is likely due to the fact that mental evocation acts as a simulation of reality (Ji et al., 2016); when engaging in this process, individuals experience the objects, situations, or events “as if they were real”.

In studies aimed at collecting the content of SRs, participants are implicitly or explicitly asked to mentally evoke the object under investigation. They are also asked to verbalize various aspects of their mental imagery. Most SR research relies on verbal productions collected through interviews or free association tasks. It is reasonable to assume that these verbal materials may contain traces of the emotion’s participants felt at the moment they were asked to think about the object.

Research has shown that words carry relatively consistent emotional connotations across speakers (Kauschke et al., 2019; Leveau et al., 2011). Emotional valence norms have been established for extensive lists of words across different languages (Bradley & Lang, 1999; Syssau & Font, 2005; Võ et al., 2009). Furthermore, automated systems for detecting emotions in text—originally developed by Pennebaker and Francis (1999)—have become increasingly sophisticated (for overviews, see Nandwani & Verma, 2021; Zad et al., 2021). These systems operate on a shared principle: analyzing and counting emotionally connoted elements in a text corpus can help assess its emotional charge. In the context of SR research, this approach is only meaningful if we assume that the emotional charge of a given textual corpus reflects the emotions aroused in the individuals who produced it. Some studies provide an empirical support for this assumption.

For example, in a study by Lee and Wagner (2002), 54 female participants were asked to describe a recent emotional experience—either positive or negative—for two minutes. Their verbal accounts were recorded, and their facial expressions were secretly filmed. At the end of the session, participants were asked to classify the emotions they felt while recounting the experience as either positive or negative. Meanwhile, two female judges evaluated the emotional tone of each narrative and of the facial expressions (without audio). The results showed that the emotions participants reported feeling during their narratives matched the emotional nature of the events they described. Moreover, both the judges’ evaluations of the stories and of the facial expressions aligned with the emotional valence of the recounted episodes.

Tavani and Collange (2017) conducted a study in which 151 participants were asked to associate four words or short phrases with a dramatic news event that had occurred two years earlier in France.^1^ Participants then rated the connotation (positive or negative) of their own verbal associations and reported their current emotional state during data collection. The results revealed a significant correlation between the emotional connotation of the verbal associations and participants’ self-reported emotions. However, it remains unclear whether this correlation results from the emotional expression during verbalization or simply from the act of evoking the event.

Research by Mills and D’Mello (2014) offers further insight. In one study, participants were asked to write about an event that had elicited anger (vs. fear). They reported their emotional state both before and after the writing task. The written texts were then evaluated by two judges, who confirmed that the narratives were consistent with the targeted emotions (anger or fear). Results showed that participants experienced a moderate level of the target emotion before writing, followed by a significant increase afterward.

Together, these studies suggest that mental evocation of an object can elicit genuine emotional responses, and that verbalizing these evocations can reveal emotional content that corresponds with the individual’s internal state. In the context of SR research, evaluating the emotional charge of corpora produced in response to a given object may therefore serve as a valid proxy for assessing the emotional impact of that object on the participants.

## Social Representations, Emotions, and the Social Sharing of Emotions

From the early stages of the development of SR theory, several scholars have acknowledged a connection between SRs and emotions (Marková & Wilkie, 1987; Molinari & Emiliani, 1996; Moscovici, 1988; Wagner, 1992) and have even encouraged further exploration of the relationship between these two concepts (Joffe, 2002; Scherer, 1992). Since these early contributions, a significant body of theoretical and empirical work has addressed this issue. A synthesis of these studies has identified several key ways in which SRs and emotions are interconnected (Piermattéo, 2021). Broadly, research about the role of emotions in representational processes can be grouped into two main areas: the place that affect is likely to occupy within SRs (i.e., the emotional component) and the influence of emotions on the processes of SR formation and maintenance (i.e., representational dynamics).

In regard to the emotional component of SRs, Deschamps and Guimelli (2002) argued that interpersonal communication resulting from the social sharing of emotions can lead to a more enduring integration of affect into the representation of the object in question. For instance, discussing the anger one feels about a controversial law or the fear sparked by a newly emerging disease may contribute to the long-term embedding of these emotional reactions within one’s representation of the object—even though emotions are, by nature, transient (Keltner & Gross, 1999).

Lheureux and Guimelli (2009) suggested that this apparent paradox—between the fleeting nature of emotions and their durable influence on SRs—could be explained by the transformation of emotions into more stable forms such as “sentiments” (Frijda et al., 1991) or emotional attitudes (Oatley, 2000; see also Piermattéo, 2021, for a distinction between these concepts and emotions within SR theory). Research in this area has shown that specific emotional responses can be associated with different elements of representational content. For example, using self-report measures, Deschamps and Guimelli (2002) found that the word “attacks” within representations of insecurity was strongly linked to fear, while other content items were associated with emotions like anger. This affective component of SRs may also manifest through content that explicitly names emotions. For instance, Ginguené et al. (2023) identified terms such as “anger” and “sadness” in individuals’ representations of terrorism (see also Tavani & Collange, 2017, for comparable findings).

With respect to representational dynamics, Moscovici (1961, 2008) observed that the emergence of a new object in the social environment typically prompts individuals to seek out information, often through interpersonal communication, which in turn contributes to the construction of a shared SR of that object. More recent research (Bouriche, 2014, 2022; Piermattéo, 2013), however, suggests that this communicative behavior may not be driven solely by a desire for knowledge. Emotions themselves may be a powerful motivator.

Rimé (2005) and Guimelli and Rimé (2009) proposed that the appearance of a novel object—or any event that challenges one’s worldview—can generate emotional reactions, especially when perceived as threatening (Joffe, 2003). These emotional responses may prompt individuals to seek out others with whom they can share their emotions. This social sharing of emotions, in turn, can influence the dynamics of SRs by fostering interpersonal exchanges about socially significant objects and promoting the collective construction of knowledge, beliefs, and opinions (Rimé, 2020).

In line with this perspective, Bouriche (2022) proposed an integrative model that conceptualizes SRs as cognitive-emotional processes. This model underscores the essential role of emotions in representational dynamics and highlights the central importance of interpersonal communication. According to Bouriche, such communication—often involving the social sharing of emotions—is driven not only by socio-affective needs (e.g., emotional relief or “venting”), but also by epistemic motivations (e.g., engaging in dialogue to evaluate and make sense of one’s emotional experience in relation to the object).

## Research Overview

Based on the theoretical framework outlined above, we argue that the more strongly a representational object elicits emotion in an individual, the greater their need to share that emotional experience with others. For a given SR object, we therefore expect to observe a positive correlation between the frequency of interpersonal communication about the object and the intensity of the emotions it provokes. To test this hypothesis, we conducted two studies. The first, an exploratory study, focused on the social representation of AIDS. The second addressed the social representation of war.

## Study 1. AIDS SR and Discussion Partners: An Exploratory Study

This study was originally designed to explore SR of AIDS in Russia (Bovina, 2008) and did not initially focus on emotions or social sharing. However, it is particularly relevant because participants were asked not only to describe their representations of AIDS, but also to indicate the individuals with whom they occasionally discussed the topic. This aspect of the design provided a measure of social sharing comparable to those used in previous research (e.g., Duprez et al., 2015). To align the study with our current research questions, we conducted additional analyses to evaluate the emotional intensity of participants’ responses. It is important to note that data for this research were collected during a period when formal ethical review procedures (e.g., ethics committees) were not yet standard practice in social psychology research.

### Sample and Method

The study sample consisted of 294 Russian citizens (*M*_age_ = 20.92, *SD*_age_ = 4.20; 39.12% male, 60.88% female). Participants completed an online questionnaire in which they were asked to generate five words or short phrases associated with the inductive term “AIDS”. They then rated their level of concern about the issue of AIDS on a scale from 1 (*not concerned at all*) to 8 (*very much concerned*).

Finally, participants indicated with whom they sometimes discussed the topic of AIDS, selecting one or more options from the following list: parents, friends, sexual partners, colleagues, acquaintances, doctor, and other.

In the first stage of analysis, the 1,426 verbal associations collected were translated into French. To ensure translation accuracy, we used three automatic translation tools (Microsoft Translator, Google Translate, and DeepL). For each association, we retained a translation if at least two of the tools provided the same result. Using this method, 1,384 verbal associations were successfully translated. The remaining 42 associations—less than 3% of the total corpus—for which no agreement was found across at least two tools were excluded from further analysis.

The translated verbal associations were then analyzed using Tropes software, incorporating the EmotAix lexicon (Piolat & Bannour, 2009; Moliner et al., 2025). This lexicon contains 4,921 French words with emotional connotations, categorized into five types: positive emotions, negative emotions, unspecified emotions, surprise, and impassivity. The tool identifies all emotionally connoted terms within a text corpus.

In our analysis, the software identified 90 words with negative emotional connotations. However, for the purposes of this study, we included only those words that explicitly conveyed an emotion or feeling. Terms such as *catastrophe, condemned*, or *tragedy*—while emotionally charged—were excluded because they do not directly express emotional states. A refined reference list of 52 clearly emotional words was thus created (see Table 1). Each participant was then assigned an emotional score ranging from 0 to 5, based on the number of words from the reference list that appeared in their set of verbal associations.

**Table 1:** List of words used to calculate participants’ emotional scores in Study 1.


WORDS WITH EMOTIONAL CONNOTATIONS SELECTED ON THE BASIS OF THE EmotAix LEXICON			

alienation	difficulty	impotence	shame

anger	disappointment	injustice	shock

anxiety	disgrace	isolation	solitude

apathy	disgust	lack	sorrow

bitterness	doubt	loss	sorry

boredom	empty	misfortune	stress

confusion	evil	oppression	suffering

danger	exhaustion	pain	threat

dependency	fear	panic	uncertainty

depressed	fear	pessimism	unfair

depression	frightening	regret	unhappy

despair	hatred	rejection	vulnerability

desperate	horror	sadness	weakness


### Hypothesis

We hypothesized that the more participants used verbal associations expressing negative emotions, the greater the number of interlocutors with whom they would report discussing the topic of AIDS. Accordingly, we expected a positive correlation between participants’ emotional scores and the number of interlocutors indicated.

### Results

The analysis revealed an average emotional score of 1.08 (*SD* = 1.08). Notably, 36.39% of participants did not use any of the emotional words from the reference list.

A significant, though modest, positive correlation was found between the number of declared interlocutors (*M* = 1.09, *SD* = 1.36) and participants’ emotional scores, *r*(292) = .23, *p* < .001. There was also a significant correlation between participants’ level of concern about AIDS (*M* = 4.37, *SD* = 1.60) and their emotional scores, *r*(292) = .16, *p* = .006. Additionally, level of concern was positively correlated with the number of interlocutors, *r*(292) = .33, *p* < .001.

To further examine the relationship between emotion and the content of AIDS representations, we divided the sample into two subgroups. The first subgroup included 107 participants who produced none of the 52 emotional words from the reference list (see Table 1). The second subgroup consisted of 187 participants who used at least one emotional word. We then identified the words (excluding the emotional words listed in Table 1) that were mentioned by at least 10% of participants in each subgroup. Table 2 presents the frequencies of these terms.

**Table 2:** Frequency of words produced by at least 10% of participants who produced no emotional words or who produced at least one emotional word.


	NO EMOTIONAL WORDS (*n* = 107)		AT LEAST 1 EMOTIONAL WORD (*n* = 187)		χ*^2^*(1, *N* = 294)
	
	*n*	*%*	*n*	*%*	

illness	55	51.40%	83	44.39%	0.48

death	54	50.47%	88	47.06%	0.11

drugs	35	32.71%	19	10.16%	15.26***

sex	34	31.78%	26	13.90%	8.54**

blood	19	17.76%	10	5.35%	9.41**

HIV	17	15.89%	9	4.81%	8.46**

syring	15	14.02%	11	5.88%	4.59*

immunity	14	13.08%	14	7.49%	2.02

condom	11	10.28%	2	1.07%	12.23***

promiscuity	11	10.28%	7	3.74%	4.40*

incurable	10	9.35%	25	13.37%	0.84

addiction	0	0.00%	30	16.04%	*p* < .001


*Note*. *p < .05. **p < .01. ***p < .001. For “addiction” a Fisher exact test was performed.

Most of the terms for which differences emerged between the subgroups referred to modes of virus transmission (e.g., drug, sex, blood, syringe, promiscuity, and addiction). With the exception of “addiction”, these words were more frequently mentioned by participants who did not produce emotional words. Additionally, the acronym “HIV” and the word “condom” appeared more often in the non-emotional subgroup. While we will not elaborate on these differences within the broader framework of SR theory, as such analysis exceeds the scope of this article, it is worth noting that the presence of emotional connotations appears to shift attention away from virus transmission. This suggests that the emotional arousal linked to the object AIDS may alter the cognitive focus of participants’ representations.

### Discussion

The results support our initial hypothesis: participants who expressed more emotion in their verbal associations also reported a greater number of discussion partners regarding the topic of AIDS. This finding aligns with the theoretical assumption that emotional arousal can motivate interpersonal communication about representational objects. However, this study presents a notable limitation. The corpus of verbal associations was translated from Russian, while the EmotAix lexicon used for analysis was developed for texts in French. As a result, the emotional tone of the translated corpus may not fully reflect the emotions actually felt by the Russian-speaking participants. Words that carry negative emotional connotations in French may not convey the same emotional intensity in Russian. This discrepancy could help explain the relatively modest correlation observed between participants’ emotional scores and their reported number of interlocutors. To address this limitation, a second study was conducted with a French-speaking population.

## Study 2: Social Representation of War, Emotions, and Motivations Linked to the Social Sharing of Emotions

The aim of this second study was to replicate the findings from Study 1 while introducing additional elements to further explore the relationship between SRs, emotional responses, and social sharing. Specifically, we sought to investigate the motivations behind interpersonal communication related to a SR object. Such communication can be driven by a variety of motives—ranging from emotional expression to information seeking (Duprez et al., 2015).

Whereas Study 1 inferred emotional content through lexical analysis using the EmotAix lexicon, Study 2 adopted a more direct approach: participants were asked to report their emotional reactions to the representational object itself. This allowed for more precise measurement of the emotions elicited. Additionally, we explored the sources from which participants obtained information about the SR object as an exploratory component.

This study was pre-registered on the Open Science Framework (https://osf.io/u8fcv) and received ethical approval from the Ethics Committee of Université Paul-Valéry Montpellier 3 (IRB00013686-2024-17-CER UPVM).

### Sample and Method

To determine the necessary sample size, an a priori power analysis was conducted using G*Power. Based on the effect size observed in Study 1 (*f^2^* = .05), with a statistical power of 80% and α = .05, the recommended sample size was *N* = 244. A total of 248 French residents participated in the study (*M*_age_ = 44.67, *SD*_age_ = 13.16; 53.23% male, 46.77% female), completing an online questionnaire.

The questionnaire began with a free association task in which participants were asked to list five words or phrases that came to mind in response to the term “war”.

To assess the emotional component of the representation, participants were asked to rate how the object made them feel using nine 7-point Likert scales ranging from 1 (*not at all)* to 7 (*completely*). Of these nine scales, based on Yzerbyt et al. (2003), three assessed anger (annoyed, irritated, revolted), three assessed sadness (sad, depressed, despondent), and three assessed fear (frightened, anxious, terrified).

Social sharing was measured with two items. The first assessed the frequency of social sharing about the object over the past 30 days (0 = *never*, 1 = *rarely*, 2 = *fairly often*, 3 = *very often*), and the second, adapted from Duprez et al. (2015), assessed the number of social sharing partners (from 1 to “more than 5”).

To explore motivations for social sharing—recognizing that existing scales are tailored to discrete emotional events and not general SR objects—we developed an *ad hoc* instrument. Participants rated the degree to which they agreed with various motives using 7-point Likert scales ranging from 1 (*not at all*) to 7 (*completely*) in response to the prompt:“Generally speaking, when I talked about the war, it was to…”. Motivations were grouped into three theoretically informed dimensions: (a) emotional motivations (e.g., “to express my emotions,” “to relieve myself”), grounded in the literature on emotional sharing (Delfosse et al., 2004; Duprez et al., 2015); (b) epistemic motivations, based on SR theory (e.g., “to know what others were thinking,” “to get an external opinion”) reflecting the desire for knowledge and sense-making (Moscovici, 1961, 2008; Rateau et al., 2011); and (c) social-normative motivations, derived from both SR theory and social sharing frameworks (e.g., “to feel supported,” “to know whether others agree with me,” “to assess whether my opinion is socially acceptable”; Prost et al., 2023; Rimé et al., 2020).

Finally, participants were asked to indicate the sources from which they had obtained information about war (e.g., social media, television/radio, print media, scientific literature, knowledgeable persons, other). However, data from this question were not included in the current analyses.

### Hypotheses

This study tested the following hypotheses:

1. We expected a positive correlation between the emotions reported by participants and both indicators of social sharing.
2. We expected a significant positive relationship between self-reported social sharing and the different motivations for sharing. Specifically, based on prior research (Duprez et al., 2015), we hypothesized that emotional motivations would be the most prominent drivers of social sharing.

### Results

We first computed an emotional score for each participant by averaging responses to the nine emotional scales (ω = .88). The two indicators of social sharing—frequency and number of interlocutors—were significantly correlated, *r*(246) = .68, *p* < .001. Therefore, a composite social sharing score was created by summing the two. A significant positive correlation was found between the emotional score and the social sharing score, *r*(246) = .23, *p* < .001.

A principal component analysis was performed on the 18 motivation items. Three components emerged with eigenvalues greater than 1. Five items were excluded due to low factor loadings (< .40) or problematic cross-loadings (i.e., < .20 difference between primary and alternative loadings; Hinkin, 1998; Howard, 2016). The final solution included 13 items (KMO = .89; item-level KMO > .81; χ^2^(78) = 1841, *p* < .001) distributed over three components which explained 69.20% of the total variance (see Table 3). Component 1 (26.80% of variance) included five items reflecting emotional expression and support. Component 2 (25.50%) included five items reflecting epistemic motivations, such as meaning-making and information-seeking. Component 3 (16.90%) included three items associated with social bond motivations.

**Table 3:** Results from a principal component analysis of motivations for social sharing.


SOCIAL SHARING MOTIVATIONS ITEMS	FACTOR LOADING		

	1	2	3

Factor 1: Emotion expression and social support seeking			

To relieve myself.	**.81**	–.02	–.04

To be understood.	**.78**	–.00	.21

To be supported.	**.77**	–.05	.20

To express my emotions.	**.72**	.28	–.22

To be listened to.	**.70**	.15	.09

Factor 2: Social sense-making			

To find out what the other person thought about it.	–.03	**.87**	.04

To find out how the other person felt about it.	.18	**.77**	–.14

To get an outside perspective.	–.05	**.73**	.19

To reflect on this subject.	.23	**.65**	–.21

To find out what the other person and I agreed on.	.13	**.63**	.30

Factor 3: Bonding			

To arouse interest.	.09	.07	**.79**

To be taken into account.	.29	–.13	**.77**

To find out what the other person and I disagreed about.	–.06	.43	**.66**


*Note. N* = 248. The extraction method was principal component analysis with an oblique (Oblimin) rotation. Factor loadings in bold denote each item’s corresponding component.

Based on this analysis, we computed three motivational scores: “emotional expression and regulation” motivation (i.e., Factor 1, ω = .88), “epistemic” motivation (i.e., Factor 2, ω = .86) and “social bond” motivation (i.e., Factor 3, ω = .81). Each of these motivational dimensions was positively correlated with the social sharing score (*r* ranging from .27 to .43, all *p* < .001; see Table 4).

**Table 4:** Descriptive statistics and correlations regarding motivations for social sharing and the Social Sharing Indicator.


	*M*	*SD*	1	2	3	4

1. “emotional expression and regulation” motivation	3.33	1.49	–			

2. “epistemic” motivation	4.54	1.39	.65***	–		

3. “social bond” motivation	2.45	1.42	.58***	.45***	–	

4. Social sharing	5.69	1.67	.39***	.43***	.27***	–


*Note*. *** *p* < .001.

Contrary to our second hypothesis, participants reported significantly higher epistemic motivation (*M* = 4.54, *SD* = 1.39) than emotional expression and regulation motivation (*M* = 3.33, *SD* = 1.49), *t*(247) = 15.60, *p* < .001, *d* = 0.99, 95% CI [0.84, 1.14], and social bond motivation (*M* = 2.45, *SD* = 1.42), *t*(247) = 22.40, *p* < .001, *d* = 1.42, 95% CI [1.24, 1.60]. Emotional and social bond motivations also differed significantly, *t*(247) = 10.30, *p* < .001, *d* = 0.66, 95% CI [0.52, 0.79].

To further examine predictors of social sharing, we conducted a hierarchical multiple regression with the social sharing score as the dependent variable and the emotional score and three motivation types as predictors, controlling for age and gender (see Table 5).

**Table 5:** Hierarchical Regression Result for social sharing.


DEPENDANTVARIABLE	VARIABLE	*B*	95% CI FOR *B*		*SE B*	*β*	*R^2^*	Δ*R^2^*

			*LL*	*UL*				

Social sharing	Step 1						.01	.01

	Constant	5.26***	4.50	6.01	0.38			

	Sex	–0.22	–0.63	0.20	0.21	–.06		

	Age	0.01	–0.00	0.03	0.01	.09		

	Step 2						.22	.21***

	Constant	2.61***	1.52	3.69	0.55			

	Sex	–0.26	–0.64	0.13	0.20	–.08		

	Age	0.01	–0.00	0.03	0.01	.08		

	Epistemic motivation	0.35***	0.17	0.53	0.09	.29***		

	Emotional expression and regulation motivation	0.17	–0.02	0.36	0.10	.15		

	Social bond motivation	0.05	–0.13	0.21	0.09	.04		

	Emotion score	0.10	–0.08	0.27	0.09	.07		


*Note*. CI = confidence interval; LL = lower limit; UL = upper limit. Sex (0 = men, 1 = women). *p > .05. **p < .01. ***p < .001.

In Step 1, age and gender were entered as control variables. In Step 2, the emotional score and motivation scores were added. The final model explained 22% of the variance in social sharing behavior, *F*(6, 241) = 11.62, *p* < .001, η^2^ = .22. The only significant predictor was epistemic motivation (β = .29, *t* = 3.89, *p* < .001).

These findings may appear surprising. While we did find a significant, albeit modest, relationship between reported emotions and social sharing, participants identified epistemic motivations—rather than emotional ones—as the primary reason for social sharing. As shown in Table 5, epistemic motivation was the only significant predictor of social sharing in the regression model. Taken together, these results suggest that emotions related to the SR of war influence social sharing indirectly—by activating a need for understanding and information. In other words, emotional arousal seems to foster epistemic motivation, which in turn leads to communication.

To test this hypothesis, we conducted a parallel mediation analysis using PROCESS 4.1 for SPSS (Hayes, 2022; Model 4), with 10,000 bootstrap simulations. In addition to epistemic motivation, emotional motivation and social bond motivation were entered as parallel mediators. Gender and age were included as covariates.

**Figure 1:**
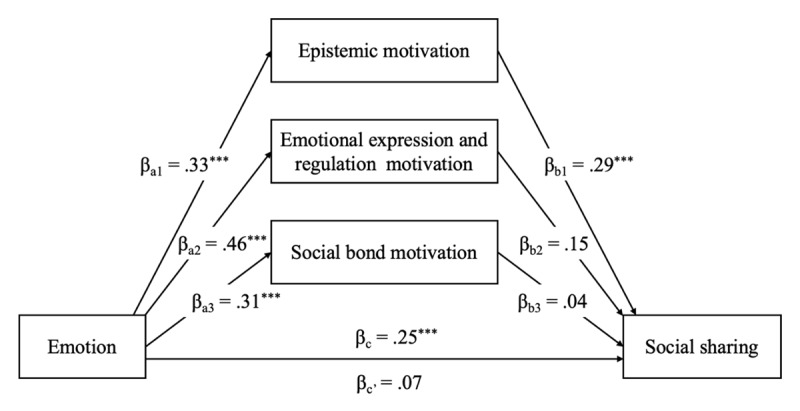
Parallel mediation: Motivations mediating the relationship between emotions associated with the social representation of war and social sharing.

Figure 1 shows that the only significant indirect effect was through epistemic motivation (βab_1_ = .10, *SE*_boot_ = .03, 95% CI [.04, .16]). The indirect effects through emotional motivation (βab_2_ = .07, *SE*_boot_ = .04, 95% CI [–.00, .15]) and social bond motivation (βab_3_ = .01, *SE*_boot_ = .02, 95% CI [–.03, .05]) were not significant. These results confirm that epistemic motivation is the sole mediator linking emotional response to social sharing behavior in this context.

To examine whether self-reported emotions influenced the content of SR, we divided the participants into two subgroups according to their emotional score. The first subgroup consists of 61 participants with emotional scores in the first quartile (emotional score < 4.11), and the second subgroup consists of 63 participants with emotional scores in the last quartile (emotional score > 5.66). We examined words mentioned by at least 10% of each subgroup. Frequencies are shown in Table 6.

**Table 6:** Frequency of words produced by at least 10% of participants by emotional score.


	EMOTIONAL SCORE < 4.11 (*n* = 61)		EMOTIONAL SCORE > 5.66 (*n* = 63)		χ*^2^*(1, *N* = 124)
	
	*n*	*%*	*n*	*%*	

death	44	72.13%	42	66.67%	0.43

weapon	17	27.87%	14	22.22%	0.52

destruction	11	18.03%	10	15.87%	0.1

violence	10	16.39%	7	11.11%	0.73

soldier	9	14.75%	5	7.94%	1.43

blood	8	13.11%	8	12.70%	0.004

fear	7	11.48%	10	15.87%	0.5

peace	7	11.48%	1	1.59%	5.02*

sadness	6	9.84%	9	14.29%	0.57

injustice	4	6.56%	8	12.70%	1.33

horror	3	4.92%	13	20.63%	6.81**


*Note*. *p < .05. ** *p* < .01.

Two significant differences emerged. First, the word “horror” was cited by 20.63% of the high-emotion group and only 4.92% of the low-emotion group, *χ^2^*(1, *N* = 124) = 6.81, *p* = .01. Additionally, the word “peace” was cited more frequently by the low-emotion group (11.48%) than by the high-emotion group (1.59%), *χ^2^*(1, *N* = 124) = 5.02, *p* < .05. Moreover, words explicitly expressing emotions (i.e., “fear”, “sadness”, “horror”) represented 10.25% of the verbal associations in the high-emotion group, compared to 5.33% in the low-emotion group. This difference was statistically significant, *χ^2^*(1, *N* = 612) = 5.12, *p* = .02. These findings suggest that participants who reported stronger emotions also more frequently integrated emotional content into their representations of war.

### Discussion

The results of this second study generally support the findings from Study 1 while offering new insights. Once again, we found that the intensity of emotional arousal related to a representational object is positively associated with social sharing behavior.

However, the study also shows that emotions alone do not directly explain why people engage in interpersonal communication about the object. Instead, participants primarily shared because they sought to understand, interpret, and process the object—highlighting the central role of epistemic motivation. Mediation analysis confirmed that emotional arousal contributes to social sharing indirectly, via the activation of epistemic needs. This shift in motivation has theoretical implications for the study of SRs and social sharing: it underscores that emotion may not trigger social interaction for expressive reasons alone, but also because it creates uncertainty, or ambiguity that individuals try to resolve through dialogue and meaning-making.

## General Discussion

In line with previous work by Rimé (2005, 2020) and the findings of Bouriche (2014), the results from both studies demonstrate a consistent relationship between the emotional charge of SRs content and individuals’ self-reported social sharing behavior. This pattern was observed across two different representational objects—AIDS and war—and in two distinct populations (Russian and French all-comers). While the observed correlations were modest in magnitude, their consistency across studies suggests a degree of robustness in the phenomenon under investigation.

A methodological limitation of both studies, however, lies in the reliance on self-reported, retrospective measures of social sharing. As Rimé (2009) noted, such measures may not fully capture the complexity or immediacy of social sharing episodes. Furthermore, the correlational nature of the findings precludes strong causal inferences. Future research employing real-time or longitudinal designs could help address this limitation and clarify the causal relationship between associating emotions with a SR object and interpersonal communication.

Despite these limitations, our findings provide valuable insights into the role of emotions in SR processes, particularly through the lens of epistemic motivation. Study 2 revealed that while emotional intensity correlated positively with social sharing, participants were primarily motivated to engage in interpersonal communication by a desire to understand, evaluate, or clarify their representations—what we termed epistemic motivation. This aligns with Bouriche’s (2022) proposal that the emotional dynamics of SRs involve not only socio-affective processes but also cognitive ones, particularly when individuals encounter uncertainty.

To fully grasp the implications of this finding, it is essential to differentiate between motivations for social sharing following emotionally experienced events (as classically studied in the social sharing of emotions paradigm) and motivations following the evocation of a representational object. Rimé et al. (1998, 2020) have emphasized that social sharing typically serves both socio-affective and cognitive functions. While socio-affective motives—such as emotional relief or support—often predominate, especially in response to personally lived emotional episodes (Duprez et al., 2015), cognitive or epistemic motives may come to the forefront in other contexts. Indeed, as prior research suggests (Delfosse et al., 2004; Luminet et al., 2000), the hierarchy of these motives is fluid and context-dependent.

Our data point to a unique pattern in the context of SRs: the evocation of an SR object—especially one not directly experienced but mediated through media or cultural discourse—may elicit emotions that are lower in intensity. In Study 2, only 6.45%^2^ of participants mentioned a specific conflict when prompted with the word “war”, indicating a predominantly generic or abstract engagement with the concept.

This difference likely explains the predominance of epistemic motivation observed in our results. Compared to studies in which participants recalled vividly experienced emotional events—such as the study by Duprez et al. (2015), in which emotional scores exceeded 5 on a 7-point scale—our participants reported more moderate levels of emotional activation (*M* = 4.84 in Study 2; *M* = 1.26 in Study 1). It is plausible that such moderate emotional states do not elicit the same need for catharsis or social support, but instead stimulate cognitive uncertainty, leading to efforts to construct meaning through interpersonal discussion.

Thus, in the context of SRs, we propose that emotional arousal acts less as a trigger for emotional expression and more as an alert signal—one that activates a need to resolve ambiguity and understand social meaning. For individuals who directly experience emotionally charged events related to the SR object (e.g., war), socio-affective motivations may play a more prominent role. Similarly, as Rimé (2005), Guimelli and Rimé (2009), and Bouriche (2022) have argued, socio-affective motivations may surface when individuals face information that challenges their existing representations or worldviews.

## Conclusion

Taken together, our findings help clarify the role of emotions in the formation and maintenance of SRs. We propose that the mental evocation of an emotionally charged SR object prompts social sharing primarily when it reactivates cognitive uncertainty. From this perspective, emotions function less as an end in themselves and more as signals of representational instability, prompting individuals to seek interaction with others in order to refine or stabilize their understanding of the object.

This raises new and important questions: Why are some individuals more sensitive to such emotional “alerts” than others? What factors moderate the transition from emotional arousal to epistemic motivation and social communication? At present, the empirical literature on social sharing in the context of SRs remains too limited to answer these questions. Future research should investigate individual differences, cultural norms, and contextual variables that shape these dynamics.

The relevance of this topic is likely to increase in the years to come. First, the rise of social media has significantly accelerated the pace and scale of social sharing (Rodríguez Hidalgo et al., 2015), making it a central mechanism in the diffusion and transformation of SRs. Second, contemporary SR objects—such as the COVID-19 pandemic, the war in Ukraine, global climate change, and geopolitical conflicts—are often introduced into public discourse through emotionally charged images. As Höijer (2010, 2011) has shown, media representations are powerful vehicles for emotional anchoring, helping to structure how individuals interpret and remember these events.

Together, these factors suggest that emotions and epistemic needs may increasingly interact to shape representational dynamics, influencing not only how individuals perceive their social world, but also how societies as a whole respond to emerging issues. The continued integration of research on SRs and social sharing of emotions appears essential for understanding these evolving processes.

## Data Accessibility Statement

The data that support the findings of this study are available at: https://osf.io/97utx.

## Footnotes

The event described was the street murder of a young anti-fascist militant, Clément Méric.

Because these responses have a low frequency (i.e., less than 10% of the corpus), they do not appear in Table 6.
